# Surgical resection of micronodular thymic carcinoma with lymphoid hyperplasia: a case report

**DOI:** 10.1186/s44215-023-00035-4

**Published:** 2023-05-03

**Authors:** Naoki Miyamoto, Kazuya Kondo, Yoshimi Bando, Fuyumi Izaki, Taihei Takeuchi, Hiroyuki Sumitomo, Shinichi Sakamoto, Mika Takashima, Naoya Kawakita, Hiroaki Toba, Hiromitsu Takizawa

**Affiliations:** 1 Department of Thoracic, Endocrine Surgery and Oncology, Tokushima University Graduate School of Medical Science, 3-18-15, Kuramoto-cho, Tokushima, 770-8503 Japan; 2 Department of Pathology, Tokushima University Hospital, Tokushima, Japan

**Keywords:** Dysmorphism, Mediastinal tumor, Median sternotomy, Thymic carcinoma, Thymoma

## Abstract

**Background:**

Micronodular thymic carcinoma with lymphoid hyperplasia is an extremely rare thymic tumor, exhibiting a variety of cell morphologies with mild to severe dysmorphism. Since few cases have been reported, the prognosis of this disease is unclear.

**Case presentation:**

A 55-year-old woman was referred to our hospital with an anterior mediastinal tumor. She was incidentally detected with a tumor in a medical examination. We diagnosed the patient with thymic carcinoma or thymoma and performed surgery via median sternotomy. Histologically, tumor cells showed weakly acidic vesicles and bright nuclei, including small nucleoli. Most of the tumor cells were cluster of differentiation (CD)5-positive, CD3-negative, and terminal deoxynucleotidyl transferase (TdT)-negative.

**Conclusions:**

Based on these histological findings, the resected specimen was diagnosed as micronodular thymic carcinoma with lymphoid hyperplasia. The patient’s postoperative course was uneventful, and no signs of recurrence were observed at 5 years after the surgery.

## Background

Micronodular thymic carcinoma with lymphoid hyperplasia (MNC) is a thymic tumor characterized by lymphoid stroma and malignant components. Since MNC is extremely rare and its histological presentation is diverse, very few reports on MNC are currently available. Herein, we present the case of a patient diagnosed with MNC.

## Case presentation

**Fig. 1:**
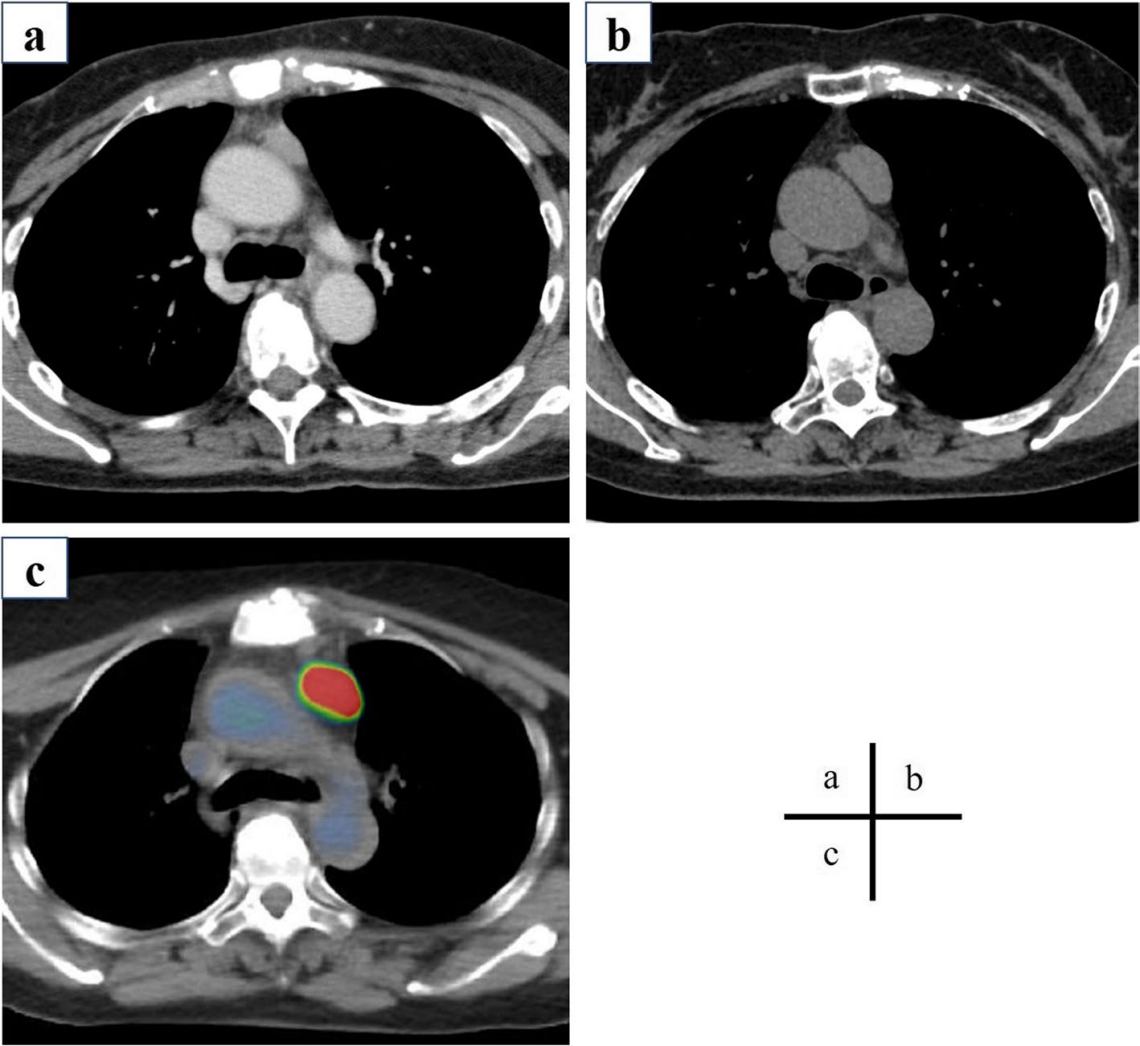
Chest computed tomography revealed a tumor in the anterior mediastinum (**a**). The tumor enlarged over a period of 2 years (**b**). PET-CT imaging revealed an FDG accumulation in the tumor (**c**). PET-CT positron emission tomography with computed tomography, FDG fluorodeoxyglucose

**Fig. 2:**
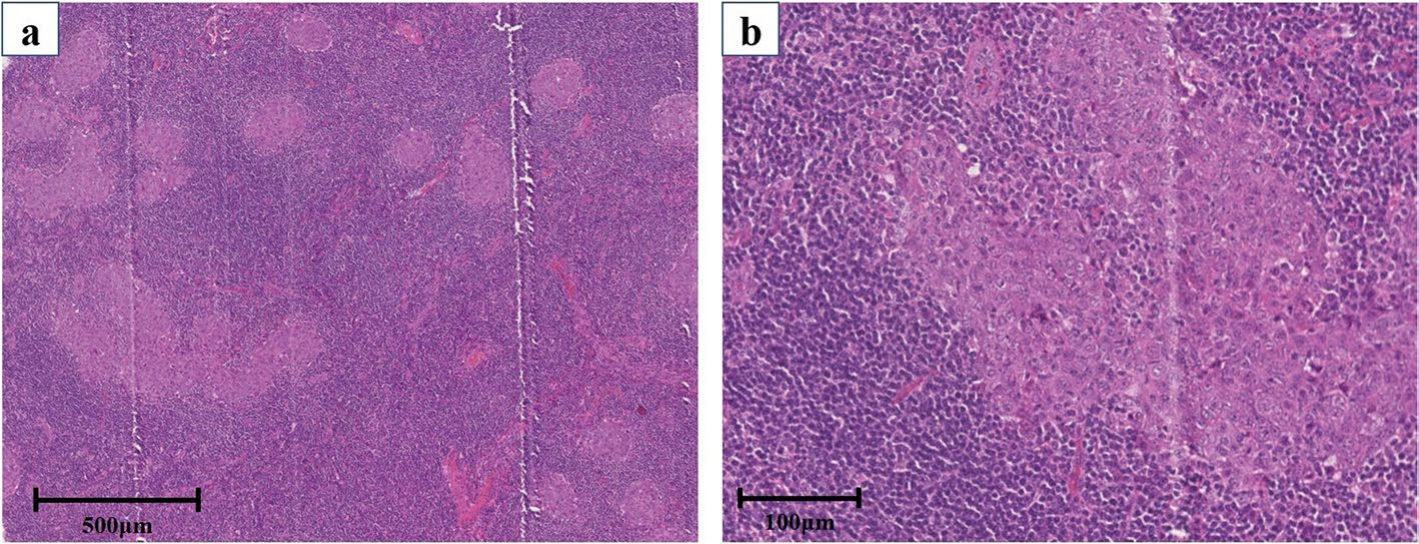
Histological examination revealed that the tumor consisted of small lymphocytes and island-shaped epithelioid cell follicles (**a**). The nuclei of epithelioid cells were oval and relatively large (**b**)

**Fig. 3:**
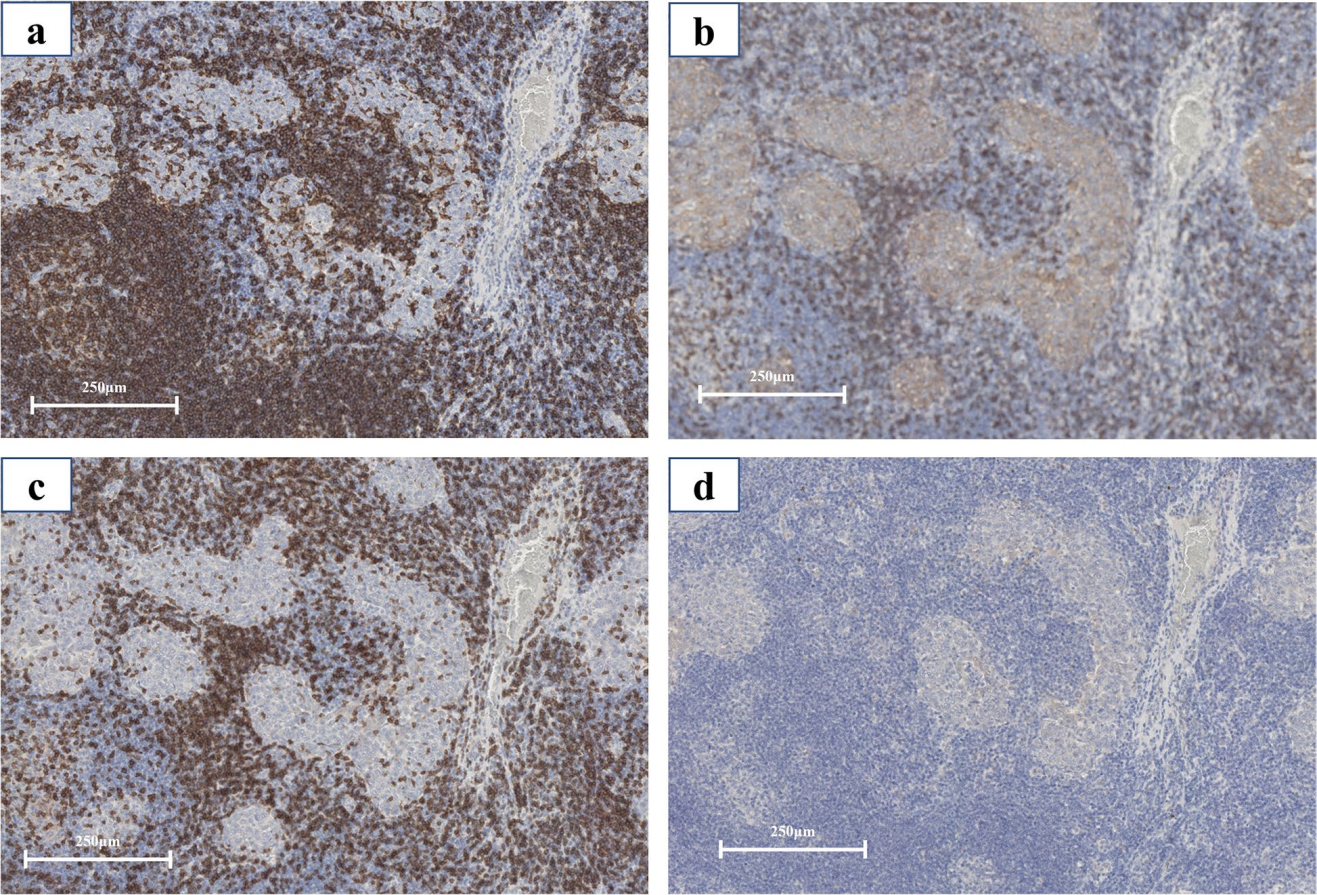
Histological immunostaining: lymphoid follicles were CD20-positive (**a**). Most tumor cells were CD5-positive (**b**), CD3-negative (**c**), and TdT-negative (**d**). CD cluster of differentiation, TdT terminal deoxynucleotidyl transferase

A 55-year-old woman was referred to our hospital with an anterior mediastinal tumor. She was incidentally detected with the tumor in a medical examination. Chest computed tomography (CT) showed a 24-mm tumor in the anterior mediastinum (Fig. 1a). Since the patient had no dysphagia, dysarthria, or muscle weakness, she was followed up. The tumor size enlarged to 32 mm in 2 years (Fig. 1b). However, no abnormalities in blood biochemical findings were observed. The tumor markers, alpha-fetoprotein ([AFP], 5 ng/mL) and β-human chorionic gonadotrophin ([β-hCG], 0.88 ng/mL), were not elevated. Tumor positron emission tomography and CT (PET-CT) using F18-fluorodeoxyglucose (F18-FDG) revealed FDG accumulation and a maximum standardized uptake value of 25.2 by the tumor (Fig. 1c). Hence, we diagnosed the patient with thymic carcinoma or thymoma and performed surgery via median sternotomy. The tumor did not invade the surrounding area, and extended thymectomy was performed successfully. Histologically, the nodule in the resected specimen was covered with a capsule and consisted of small lymphocytes and island-shaped epithelioid cell follicles (Fig. 2a). The nuclei of the epithelioid tumor cells were relatively large, and anisonucleosis and irregular edges were mild (Fig. 2b). Tumor cells contained weakly acidic vesicles and bright nuclei, including small nucleoli. Mitotic figures were seen in 2–3/10 high-power fields (HPF). Ki-67 expression in tumor nests was among 1–5%, and tumor nodules were separated by abundant lymphoid stroma. The surrounding lymphoid stroma was composed of dense proliferation zones of small lymphocytes and contained some lymphoid follicles with germinal centers. Lymphocytes in the lymphoid stroma were a cluster of differentiation (CD)20-positive (Fig. 3a) and B-cell lymphoma 2 (Bcl2)-negative. Most of the tumor cells within the lymphoid stroma were CD5-positive (Fig. 3b), CD3-negative (Fig. 3c), and terminal deoxynucleotidyl transferase (TdT)-negative (Fig. 3d). Based on these histological findings, the resected specimen was diagnosed as MNC (pT1N0M0 stage I, Masaoka II). Since the tumor had been completely resected, we determined postoperative adjuvant chemotherapy as unnecessary. The patient’s postoperative course was uneventful, and the patient was discharged 7 days postoperatively. No signs of recurrence were observed at 5 years after the surgery.

## Discussion and conclusions

MNC is a rare thymic tumor characterized by lymphoid aggregates and germinal centers. Unlike most thymic tumors that are characterized by T lymphocytic infiltration, MNC forms germinal centers within the thymic cortex with a heavy infiltration of B lymphocytes. Micronodular thymomas with lymphoid stroma (MNT) were first described in 1999^1^, and MNC was more recently described as a malignant MNT counterpart^2^. These two types of tumors are collectively referred to as micronodular thymic tumors. The incidence of MNT is as low as approximately 5% of all thymoma cases, and the incidence of MNC is even lower. A PubMed search for publications in English using the keyword “Micronodular thymic carcinoma” identified a total of 13 patients with MNC^2-6^. In these reports, the patients’ age range was 42–78 years; 8 patients were males and 5 were females. Among the 10 cases, eight cases mentioned tumor size of 1.1–10 cm, and no patient underwent a preoperative PET/CT scan. The treatment employed for twelve cases was surgical resection. Follow-up data were available for eleven cases, as one patient died of recurrence. Histologically, MNC displays a benign micronodular component similar to that of MNT, as well as a cancerous component. Regarding our case, an MNT diagnosis was excluded based on the following reasons: [1] most tumor cells showed epithelioid morphology rather than spindle shape and [2] cell atypia and mitotic figures were easily identified.

Wang et al. established the criteria for the classification of micronodular thymic tumors^4^. They mentioned the following features of MNC: [1] tumor cells with moderate-to-severe dysplasia, [2] tumor cell mitotic figures > 2/10 HPF, [3] evidence of neoplastic necrosis, [4] no terminal deoxynucleotidyl transferase-positive immature T lymphocytes within the tumor, [5] tumor cells with a Ki-67 index ≥ 10%, and [6] tumor cells expressing CD5. In the present case, the Ki-67 index was less than 10%, but the tumor cell mitotic figures were approximately 2–3/10 HPF, and the tumor cells were expressing CD5, hence pointing to a diagnosis of MNC, based on the above criteria. The number of MNC cases reported is very small, and MNC is only mentioned as a differential diagnosis of MNT in the thymoma WHO classification 4th edition [7]. Moreover, MNC can exhibit a variety of cell morphologies, ranging from mild to severe dysmorphism. Although only one out of the 10 reported patients with MNC died, the prognosis of MNC remained unclear. The case presented here is considered a low-malignant type MNC with the following features: [1] no evident tumor necrosis and [2] a slight increase in mitotic figures.

In this case, we performed extended thymectomy via a median sternotomy. The fat around the resected thymus gland contained lymph nodes, but no lymph nodal metastasis was detected. There are no reports demonstrating the benefit of lymph node dissection or postoperative adjuvant chemotherapy for thymic epithelial tumors. Therefore, we consider that performing these procedures has little significance. At the time of this surgery, our surgical choice was a median sternotomy for thymic epithelial tumors and video-assisted thoracoscopic surgery (VATS) for small benign mediastinal tumors. In recent years, the use of VATS and robot-assisted thoracic surgery (RATS) for thymic epithelial tumors has increased, and their usefulness is reported [8, 9]. These surgeries are considered acceptable if complete resection can safely be performed. Although the tumor was completely resected and no recurrence occurred, preoperative PET-CT showed high FDG accumulation; therefore, careful follow-up was deemed necessary.

## Data Availability

The datasets used and/or analyzed during the current study are available from the corresponding author on reasonable request.

## Abbreviations

AFP: Alpha-fetoprotein
Bcl2: B-cell lymphoma 2
CD: Cluster of differentiation
CT: Computed tomography
FDG: Fluorodeoxyglucose
HPF: High-power field
MNC: Micronodular thymic carcinoma with lymphoid hyperplasia
MNT: Micronodular thymomas with lymphoid stroma
PET: Positron emission tomography
TdT: Terminal deoxynucleotidyl transferase
WHO: World Health Organization
β-hCG: β-Human chorionic gonadotrophin.

## References

[1] Suster S, Moran CA. Micronodular thymoma with lymphoid B-cell hyperplasia: clinicopathologic and immunohistochemical study of eighteen cases of a distinctive morphologic variant of thymic epithelial neoplasm. Am J Surg Pathol. 1999;23:955–62. 10435566 10.1097/00000478-199908000-00014

[2] Weissferdt A, Moran CA. Micronodular thymic carcinoma with lymphoid hyperplasia: a clinicopathological and immunohistochemical study of five cases. Mod Pathol. 2012;25:993–9. 22388764 10.1038/modpathol.2012.40

[3] MneimnehWS G-PY, Kesler KA, Loehrer PJ Sr, Badve S. Micronodular thymic neoplasms: case series and literature review with emphasis on the spectrum of differentiation. Mod Pathol. 2015;28:1415–27. 26360499 10.1038/modpathol.2015.104

[4] Wang B, Li K, Song QK, Wang XH, Yang L, Zhang HL, et al. Micronodular thymic tumor with lymphoid stroma: a case report and review of the literature. World J Clin Cases. 2019;7:4063–74. 31832410 10.12998/wjcc.v7.i23.4063 PMC6906565

[5] Tateyama H, Saito Y, Fujii Y, Okumura M, Nakamura K, Tada H, et al. The spectrum of micronodular thymic epithelial tumours with lymphoid B-cell hyperplasia. Histopathology. 2001;38:519–27. 11422495 10.1046/j.1365-2559.2001.01133.x

[6] Liu PP, Su YC, Niu Y, Shi YF, Luo J, Zhongcorresponding DR. Comparative clinicopathological and immunohistochemical study of micronodular thymoma and micronodular thymic carcinoma with lymphoid stroma. J Clin Pathol. 2022;10:702–5. 10.1136/jclinpath-2021-207819 PMC9510435 34493600

[7] Tateyama H, Marx A, Strobel P, et al. Micronodular thymoma with lymphoid stroma. In: Travis WD, Brambilla E, Burke AP, Marx A, Nicholson AG, editors. WHO classification of tumours of the lung, pleura, thymus and heart. 4th ed. Lyon: International Agency for Research on Cancer; 2015. p. 205–6.

[8] Xie A, Tjahjono R, Phan K, Yan TD. Video-assisted thoracoscopic surgery versus open thymectomy for thymoma: a systematic review. Ann Cardiothorac Surg. 2015;6:495–508. 10.3978/j.issn.2225-319X.2015.08.01 PMC4669250 26693145

[9] Shen C, Li J, Li J, Che G. Robot-assisted thoracic surgery versus video-assisted thoracic surgery for treatment of patients with thymoma: a systematic review and meta-analysis. Thorac Cancer. 2022;2:151–61. 10.1111/1759-7714.14234 PMC8758429 34806328

